# Association between the early or late onset of gestational diabetes mellitus with neonatal adverse outcomes: a retrospective cohort study

**DOI:** 10.1186/s40842-024-00196-3

**Published:** 2024-12-12

**Authors:** Fabiana Vieira Duarte de Souza Reis, Carlos Izaias Sartorão Filho, Luis Sobrevia, Caroline Baldini Prudencio, Bruna Bologna, Luana Favaro Iamundo, Adriely Magyori, Luiz Takano, Raissa Escandiussi Avramidis, Rafael Guilen de Oliveira, Marilza Vieira Cunha Rudge, Angélica Mércia Pascon Barbosa, Diamater Study Group, Carlos Izaias Sartorão Filho, Caroline Baldini Prudencio, Luana Favaro Iamundo, Luiz Takano, Raissa Escandiussi Avramidis, Rafael Guilen de Oliveira, Marilza Vieira Cunha Rudge, Angélica Mércia Pascon Barbosa, I. M. P. Calderon, F. P. Souza, T. Lehana, C. F. O. Graeff, C. G. Magalhães, R. A. A. Costa, S. A. M. Lima, M. R. K. Rodrigues, S. L. Felisbino, W. F. Barbosa, F. J. Campos, G. Bossolan, J. E. Corrente, H. R. C. Nunes, J. F. Abbade, P. S. Rossignoli, C. R. Pedroni, Á. N. Atallah, Z. I. Jármy-Di Bella, S. M. M. Uchôa, M. A. H. Duarte, E. A. Mareco, M. E. Sakalem, N. M. Martinho, D. G. Bussaneli, M. I. G. Orlandi, C. Pascon, T. D. Dangió, C. V. C. Rudge, F. Piculo, G. M. Prata, C. N. F. Carvalho, A. B. M. Magyori, G. T. A. Nava, T. C. D. Caldeirão, R. H. L. Shetty, D. R. A. Reyes, F. C. B. Alves, J. P. C. Marcondes, M. L. S. Takemoto, F. A. Pinheiro, S. B. C. V. Quiroz, T. Pascon, S. K. Nunes, B. B. Catinelli, F. V. D. S. Reis, S. M. B. Costa, M. O. Menezes, N. J. Santos, E. M. A. Enriquez, A. M. Carr, G. A. Garcia, H. C. M. Bassin, V. P. Barbosa, M. Jacomin, A. J. B. Silva, I. O. Lourenço, J. Marostica de Sá, I. P. Caruso, L. T. Rasmussen, V. K. C. Nogueira, J. T. Ribeiro-Paes, D. C. H. França, H. V. M. Bastos, M. L. A. Heliodoro, M. N. Kuroda, H. L. Carvalho

**Affiliations:** 1 Postgraduate Program on Tocogynecology, Botucatu Medical School, São Paulo State University (Unesp), Botucatu, Brazil; 2 Medical School, Educational Foundation of the Municipality of Assis, FEMA, Assis, Sao Paulo, Brazil; 3 NHMRC Centre for Clinical Research Excellence in Spinal Pain, Injury and Health, School of Health and Rehabilitation Sciences, The University of Queensland, Brisbane, Australia; 4 Departamento de Fisioterapia, Universidade Federal de São Carlos, São Carlos, Brasil; 5 Department of Physiotherapy and Occupational Therapy, Universidade Estadual Paulista (UNESP), Marília, Brazil

**Keywords:** Gestational diabetes mellitus, Neonatal outcome, Adverse pregnancy outcomes, Glucose tolerance test

## Abstract

**Background:**

The literature has been evolving to standardize gestational diabetes mellitus (GDM) diagnosis and terminology. The significance of timing in diagnosing hyperglycemia during pregnancy is underlined by evidence that women diagnosed at 24 weeks of gestation or earlier are at a higher risk of developing postpartum prediabetes, but its association with adverse outcomes for the newborn is controversial. We aimed to investigate the association between early-onset GDM and adverse outcomes in newborns and neonates, comparing it with the late-onset GDM model.

**Methods:**

It was a retrospective cohort study conducted at the Perinatal Diabetes Research Center in Assis/SP, affiliated with the Botucatu Medical School-UNESP in Brazil. The group composition was as follows: early-onset participants had fasting glucose levels ≥ 92 mg/dL and < 126 mg/dL before 20 weeks of gestation, while late-onset participants had a negative first-trimester screening and a positive 75g-OGTT at 24–28 weeks. For early-onset GDM, a fasting glucose level of ≥ 92 mg/dL is a recognized threshold associated with an increased risk of adverse pregnancy outcomes, while < 126 mg/dL ensures the exclusion of overt diabetes. The criteria for late-onset GDM, involving a negative initial screening and a positive OGTT at 24–28 weeks, align with the standard diagnostic timeframe when insulin resistance typically peaks due to placental hormone secretion. The maternal baseline characteristics included pre-pregnancy body mass index (BMI; kg/m2) and pregnancy weight gain (kg), calculated as the difference between the final pregnancy weight (36 gestational weeks or more) and pre-pregnancy maternal weight, classified according to the pre-pregnancy BMI. Additionally, the perinatal outcomes evaluated in this study included gestational age (GA) at birth, birth weight (BW) categorized according to GA as adequate, large, or small and Apgar scores at the first and 5th minutes.

**Results:**

Eight hundred eighty pregnant women were selected, of whom 203 (23.07%) presented GDM and were eligible from December 2016 to December 2021. Based on the timing onset of GDM, 89 (43.8%) were in the early-onset group, and 114 (56.2%) were in the late-onset group. The fasting plasma glucose values in the first trimester were higher in the early-onset group. The 75-g OGTT values were higher in the late-onset group. The final BMI was higher in the early-onset group. Univariate linear regression was performed to determine the relationship between late-onset and maternal and neonatal outcomes; however, no significant relation was detected.

**Conclusion:**

Pregnant women with early-onset GDM showed a higher BMI during pregnancy, but there was no difference between early and late-onset GDM concerning neonatal adverse outcomes.

## Introduction

The diagnosis of Gestational Diabetes Mellitus (GDM) had been based on hyperglycemia first recognized during pregnancy for many years. Nowadays, the American Diabetes Association (ADA) classifies GDM as diabetes diagnosed in the second or third trimester of pregnancy that was not overt diabetes before gestation or other types of diabetes occurring throughout pregnancy, such as type 1 diabetes [1]. Over the past years, studies have demonstrated that the GDM diagnosis strategy considers either first-trimester hyperglycemia or second-trimester 75g oral glucose tolerance test (75g-OGTT) screening and diagnosis [2, 3].

It is well known that the presence of GDM represents a potential risk for spontaneous abortion, fetal abnormalities, preeclampsia, fetal loss, macrosomia, neonatal hypoglycemia, hyperbilirubinemia, and neonatal respiratory distress syndrome, among other complications [1]. In the long term, it may also increase the likelihood of obesity, hypertension, and type 2 diabetes in the offspring [1].

Recent research is focused not only on the presence or absence of GDM but also on the importance of the timing of diagnosis (early-onset) [4, 5] and the intensity of hyperglycemia [6]. A systematic review revealed that macrosomia, shoulder dystocia, perinatal mortality, insulin use, and neonatal hypoglycemia were more prevalent in early-onset compared to late-onset GDM, despite treatment [4, 5]. Additionally, women diagnosed at 24 weeks of gestation or earlier are at a higher risk of developing postpartum prediabetes [7, 8]. Concerning glycemic intensity, pregnant women with higher levels of hyperglycemia had a greater chance of developing hypertensive disorders of pregnancy, preterm birth, neonatal hyperbilirubinemia, and macrosomia [6].

Recognizing pregnancy risk factors related to the timing of GDM diagnosis may positively impact the health of both the fetus and the newborn, primarily due to divergences in GDM screening and diagnosis protocols [9]. However, insufficient data exist to report the extent to which the first-trimester recognition of hyperglycemia or the conventional 24–28 weeks of gestation diabetes screening during pregnancy leads to unequal adverse outcomes for newborns [10]. The results on these topics are still preliminary, and their association with adverse outcomes for both maternal and neonatal health is poorly understood [9].

Therefore, we aim to investigate the association between early-onset GDM and adverse outcomes in neonates, comparing it with the late-onset GDM model. We hypothesize that the early-onset of hyperglycemia during pregnancy may lead to worse neonatal outcomes.

## Method

It is a retrospective cohort study at the Perinatal Diabetes Research Center (PDRC)-Assis affiliated nucleus from Botucatu Medical School-UNESP, Brazil, conducted between 2016 and 2019, approved by the Institutional Ethical Committee of Botucatu Medical School of Sao Paulo State University (Protocol Number CAAE 82225617.0.0000.5411). Pregnant women were monitored throughout pregnancy and the neonatal period. The original study design was centered around the primary objective of developing a predictive model for postpartum urinary incontinence (UI) in women who had gestational diabetes mellitus (GDM) during their pregnancy [11]. This ongoing study protocol includes all pregnant women receiving prenatal care at the obstetric and maternal–fetal medicine clinics at PDRC. We have decided to retrospectively investigate the newborn outcomes in the GDM group within this cohort, based on the onset period of hyperglycemia.

Prior to enrollment, each woman received a comprehensive explanation about the study and subsequently signed an informed consent form. All subjects met the following inclusion criteria: Patients from the antenatal care service who underwent gestational diabetes mellitus (GDM) screening and diagnosis based on WHO guidelines. Pregnant women were ineligible if they had: Multiple pregnancies, fetal loss before 22 weeks of gestation, stillbirth, loss to follow-up before data collection during pregnancy or the neonatal period, severe maternal or fetal comorbidities unrelated to GDM status during pregnancy or the perinatal period, and pre-pregnancy diabetes mellitus (DM).

Concerning patient follow-up: Following WHO guidelines, all included women initiated prenatal care before 20 weeks of gestation and underwent universal screening with fasting glucose (FG) during the first prenatal visit. For those with a negative screening, the 75g oral glucose tolerance test (OGTT) was administered in the second trimester (24–28 weeks of gestation), except for those who had undergone bariatric surgery, for whom screening was conducted with FG in the second trimester. GDM likely reflects the significant changes in maternal insulin sensitivity and hormonal dynamics during pregnancy, especially after 20 weeks, so the group composition follows:

- Early-onset: Patients with fasting glucose (FG) levels ≥ 92 mg/dL and < 126 mg/dL before 20 weeks of gestation.
- Later-onset: Patients with a negative first-trimester screening and a positive result in the 75g oral glucose tolerance test (OGTT) conducted at 24–28 weeks of gestation.

The maternal baseline characteristics considered in this study included age, parity, ethnicity (Caucasian or non-Caucasian), educational level (higher or non-higher), history of previous bariatric surgery, physical activity during pregnancy (considered positive when exceeding 150 min per week throughout the gestational period), pre-pregnancy body mass index (BMI in Kg/m^2^), and pregnancy weight gain (Kg). Pregnancy weight gain was calculated as the difference between the final pregnancy weight (at 36 weeks gestation or later) and the pre-pregnancy maternal weight, and it was categorized based on the pre-pregnancy BMI [12].

The outcomes of interest in this study include mode of delivery, preterm or term birth (gestational age at delivery), birth weight (BW) in grams, birth weight adequacy for gestational age at birth (categorized as adequate, large, or small for gestational age), Apgar scores at 1st and 5th minutes, Apgar score less than 7 at the 5th minute, and the necessity of neonate hospitalization during the first 28 days after childbirth.

Women in this study received prenatal care in our hospital’s obstetric and maternal–fetal medicine clinics. Pregnant women with gestational diabetes mellitus (GDM) received nutritional counseling through a centralized office, where they were provided instructions regarding their diet and recommended weight gain based on their pre-pregnancy BMI. Self-monitoring of plasma glucose was advised four times daily, with targets set at a fasting value of less than 95 mg/dL and two-hour post-meal values of less than 130 mg/dL. Any incomplete or missing antenatal and perinatal data were retrieved from the institutional medical records.

For the sample size calculation, we assumed a risk of an outcome event equal to 0.20 among the group with a prior diagnosis and increased the risk of an event outcome to 0.35 in the group with a late diagnosis. The ratio of the number of participants in the group with no previous obesity to those with previous obesity was set at 1:1. Type I and II errors were fixed at 0.05 and 0.20, respectively. Additionally, a maximum of three other variables were considered in the adjusted models. Based on these assumptions, it was estimated that 170 participants per group were necessary, with an estimated statistical power of approximately 0.87.

Confounding variables considered in this study included maternal age, parity, previous vaginal or C-section delivery, ethnicity, educational level, physical activities during and after pregnancy, maternal weight, previous bariatric surgery, pre-pregnancy BMI, and final BMI at delivery. Data collection procedures and statistical analysis followed the predefined period and inclusion/exclusion criteria. Data were entered into a dedicated software spreadsheet, subsequently audited, and subjected to consistency checks.

The Mann–Whitney test was employed for numerical variables, and the Chi-square test was used for categorical variables to compare groups based on the outcomes. Univariate logistic regression was conducted to estimate the relative risks (RR) and their respective 95% confidence intervals (CI) for newborn outcomes according to clinical and demographic characteristics. Subsequently, multivariate regression analysis was performed to identify factors independently associated with newborn adverse outcomes and estimate the adjusted RR (adj RR). Variables were individually inserted, and those with a *P*-value under 0.05 were retained. Cases lost to follow-up occurred entirely at random and were excluded. Additionally, missing data were sparse and occurred randomly; the imputation method addressed the few cases statistically. The analysis was conducted using SPSS version 23.0 (IBM, New York), and all statistical significances were two-sided, accepted at *P* < 0.05.

## Results

Eight hundred eighty pregnant women were screened, of which 203 (23.1%) with GDM were deemed eligible from December 2016 to December 2021. Based on the timing of GDM onset, 89 (43.84%) were classified in the early-onset group, and 114 (56.16%) in the late-onset group. Table 1 summarizes maternal characteristics. Concerning the criteria used to stratify the groups, as expected, the first trimester FPG levels were higher in the early-onset group, while the late-onset group exhibited elevated values during the 75-g OGTT. In terms of maternal health status, pregnant individuals meeting the criteria for early-onset GDM had a final BMI higher than that of the late-onset group (*p* = 0.036).

**Table 1:** Maternal characteristics according to the timing GDM onset

	**Early-onset (*n* = 89)**	**Late-onset (*n* = 114)**	
**Variables**	**n (%)**	**Med (IQR)**	p*
Non-Caucasian	14 (15.7%)	13 (11.4%)	*0.409*
Higher educational level	55 (61.8%)	73 (64%)	*0.771*
C-section	88 (98.9%)	112 (98.2%)	*1.000*
Previous bariatric surgery	0 (0%)	1 (0.9%)	*1.000*
Obesity pre-pregnancy	30 (33.7%)	26 (22.8%)	*0.113*
	**Med (IQR)**	**Med (IQR)**	
Number of gestations	2.0 (1.0–2.0)	2.0 (1.0–2.0)	*0.196*
Maternal age (years)	30.0 (26.0–34.5)	29.5 (25.0–33.3)	*0.609*
First trimester FPG (mg/dL)	95.0 (93.0–97.0)	85.0 (80.0–89.0)	*0.000*
75g OGTT (mg/dL) – fasting	83.0 (78.0–86.5)	91.7 (80.0–96.0)	*0.000*
75g OGTT (mg/dL)—1h	134.0 (113.0–150.0)	167.5 (144.0–184.0)	*0.000*
75g OGTT (mg/dL)—2h	117.0 (100.0–130.0)	140.0 (121.0–159.0)	*0.000*
BMI (kg/m^2^) pre-pregnancy	27.5 (24.1–32.1)	26.5 (23.8–29.7)	*0.202*
BMI (kg/m^2^) gestation	31.5 (28.1–36.8)	30.0 (27.4–33.7)	*0.036*

*n* sample, *g: mg/dL* miligrams per deciliter, *FPG* fasting plasma glucose, *75g OGTT* 75 g oral glucose tolerance test, *BMI* Body Mass Index; Data are presented in n(%): absolute frequency (n) and percentage (%) or Med (IQR): Median (interquartile range); *p*-values are based on *Chi-square test or Mann–Whitney U. Significance *p* < 0.05

Neonatal outcomes, as presented in Table 2, indicated similarities between early and late-onset cases regarding pre-term birth (*p* = 0.662), fetal growth restriction (*p* = 1.000), adverse neonatal outcome (*p* = 1.000), 5-min apgar < 7 (*p* = 0.633), newborn weight at birth (*p* = 0.728), 1-min apgar score (0.765) and 5-min apgar score (*p* = 0.182).

**Table 2:** Neonatal outcomes according to the GDM onset

	**Early-onset (*n* = 89)**	**Late-onset (*n* = 114)**	
**Variables**	**n (%)**	**n (%)**	p*
Pre-term birth	12 (13.5%)	12 (10.5%)	*0.662*
Fetal growth restriction	7 (7.9%)	8 (7%)	*1.000*
Adverse neonatal outcome	10 (11.2%)	12 (10.5%)	*1.000*
5-min Apgar < 7	1 (1.1%)	3 (2.6%)	*0.633*
	**Med (IQR)**	**Med (IQR)**	
Weeks of gestation	38.1 (37.3–38.9)	38.2 (37.4–38.9)	*0.732*
Newborn weight at birth (grams)	3170 (2830–3490)	3150 (2855–3400)	*0.728*
1-min Apgar score	9.0 (9.0–9.0)	9.0 (9.0–9.0)	*0.765*
5-min Apgar score	9.0 (9.0–10.0)	9.0 (9.0–10.0)	*0.182*

Data are presented in n (%): absolute frequency (n) and percentage (%) or Med (IQR): Median (interquartile range); *p*-values are based on *Chi-square test or Mann–Whitney U. Significance *p* < 0.05

Univariate linear regression, performed to determine the relationship between late-onset and various outcomes (as detailed in Table 3), did not reveal any significant associations. This suggests that there is no discernible difference in maternal outcomes, such as gestational age at delivery (*p* = 0.502), weight gain (*p* = 0.748), and c-section rates (*p* = 0.976), between participants with late or early-onset gestational diabetes mellitus (GDM).

**Table 3:** Univariate linear regression according different models, regarding gestational age, weight gain, weight on birth and Apgar > 7

**Variables**	*Β*	**95% CI**	p*
	**Investigation Model (Gestational Age)**		
Late-onset GDM	-0.16	(-0.62–0.30)	*0.502*
	**Investigation Model (Weight Gain)**		
Late-onset GDM	-22.18	(-157.46–113.09)	*0.748*
	**Investigation Model (Weight on birth)**		
Late-onset GDM	-22.18	(-157.46–113.09)	*0.748*
	**Investigation Model (Apgar > 7)**		
Late-onset GDM	8.16	(0.16–417.97)	0.296
	**Investigation Model (Pre-term birth)**		
Late-onset GDM	0.81	(0.36–1.85)	0.617
		**Investigation Model (C-section birth)**	
Late-onset GDM	1.00	(0.75–1.33)	0.976
	**Investigation Model (Fetal growth restriction)**		
Late-onset GDM	.94	(0.33–2.62)	0.899

*GDM* Gestational Diabeltes Mellitus, *BMI* Body Mass Index; Data are presented in: β: Standardized regression Coefficient; SE: Standard error; 95% CI: 95% Coeficient Interval; *p*-values are based on *Univariate linear regression significance for investigation model *p* < 0.2

Similar findings were observed in newborn outcomes, including birth weight (*p* = 0.748), Apgar scores greater than 7 (*p* = 0.296), pre-term birth (*p* = 0.617), and fetal growth restriction (*p* = 0.899). In summary, no significant differences were detected in both maternal and neonatal outcomes between individuals with late-onset GDM and those with early-onset GDM.

## Discussion

The prevalence of GDM in our study follows data from the HAPO study cohort, which ranged from 9.3% to 25.5% depending on the study site [13]. The ADA recommends a well-established protocol for GDM screening between 24–28 weeks of gestation through a 75-g OTTG [1]. The Brazilian protocol follows this recommendation but adds that pregnant with fasting glycemia from ≥ 92 to < 126 mg/dL should be diagnosed with GDM [14].

Considering this point and the fact that time of exposure and hyperglycemia intensity could be potential mediators of negative outcomes to the binomen. We decided to evaluate the impact of the early or late-onset and we verified that 43.84% of our population had an early-onset diagnosis and 56.16% a late-onset, which means that almost 50% of the population had longer exposure to hyperglycemia environment.

We observed in the early-onset GDM, a significantly higher difference in the BMI calculated at the end of the prenatal care, compared with the late-onset group. We did not find differences among the other variables studied. The relationship between early-onset GDM and obesity is complex. Obesity is a known risk factor for developing GDM, and individuals who are obese before becoming pregnant are at a higher risk of developing GDM during pregnancy. Weiss et al. reported that the GDM risk was increased 3 to 4 fold in women with obesity [15].

Pregnant individuals with obesity are at a higher risk of experiencing complications during pregnancy and childbirth. These complications can sometimes affect the newborn’s immediate health and Apgar score. For example, obese individuals are more likely to have babies with macrosomia (excessive birth weight), which can affect muscle tone and respiration in the newborn. Although our findings did not show differences between groups according to the timing of GDM diagnosis, it is important to consider this information for further studies. It is essential to highlight the new emphasis on early GDM diagnosis to prevent perinatal morbidity and mortality and identify potential long-term maternal complications. Our results evoke the importance of the concern that GDM is a clinical entity starting before pregnancy, and the crucial early-onset diagnosis may be critical for reducing adverse outcomes.

Regarding limitations, as the period for early-onset is not standardized in the literature [4] our choice of < 20 weeks as early onset and 24 weeks as late onset in the context of gestational diabetes likely reflects the significant changes in maternal insulin sensitivity and hormonal dynamics during pregnancy. Early onset at 20 weeks aligns with the onset of increasing glycaemia due to reduced insulin sensitivity. By 24 weeks, there is further elevation in glycaemia driven by placental hormonal secretion and increased insulin resistance. It could include reduced precision due to the sampling design and implementation. We also assume that it did not control how the disease and exposure variables were ascertained and recorded precisely in the pregnancy span.

Although GDM is an entity with a high prevalence worldwide and recognized association with adverse neonatal periods, we cannot fully generalize the results for different populations regarding ethnicity, socioeconomic status, parity, mode of delivery, and the plurality of variables influencing the outcome. Besides our parochial results, the presented results are critical to evoke new studies and discussions involving the screening, diagnosis, and treatment of GDM during the entire pregnancy span.

## Conclusion

Pregnant with early-onset of GDM showed higher BMI during pregnancy, but there was no difference between the early and late-onset of GDM concerning neonatal adverse outcomes. Considering clinical practice, it is important to manage obesity screening particularly in women diagnosed with GDM in early-onset. Future studies should consider including BMI as a subgroup of analysis to investigate the association between hyperglycemia, obesity, on neonatal outcomes.

## Data Availability

The authors confirm that all data underlying the findings are fully available without restriction. All relevant data is in the present manuscript.
